# Nerve Growth Factor Treatment and the Corneal Epithelium: Extracellular Vesicle‐Mediated Signaling

**DOI:** 10.1002/jex2.70192

**Published:** 2026-09-17

**Authors:** Levi N. Kanu, Amit Chatterjee, Mohamed Y. Mahmoud, Vincent Yeung, Sushma V. Mudigunda, Amy E. Ross, Brenna Hefley, Dimitrios C. Karamichos, Joseph B. Ciolino

**Affiliations:** ^1^ Department of Ophthalmology, Schepens Eye Research Institute of Mass Eye and Ear Harvard Medical School Boston Massachusetts USA; ^2^ North Texas Eye Research Institute University of North Texas Health Fort Worth Texas USA; ^3^ Department of Family Medicine, Texas College of Osteopathic Medicine University of North Texas Health Fort Worth Texas USA; ^4^ College of Biomedical and Translational Sciences University of North Texas Health Fort Worth Texas USA

**Keywords:** cornea, corneal, epithelium, exosomes, extracellular vesicles, intercellular signaling peptides and proteins, microRNAs, nerve growth factor, wound healing

## Abstract

Recombinant human nerve growth factor (NGF) is used to treat patients with impaired corneal wound healing. NGF exerts its effects through complex signaling processes initiated by the binding of one of two cell membrane receptors—TrkA and p75NTR. While the intracellular signaling pathways have been studied extensively, how NGF mediates intercellular signaling to coordinate wound healing is less understood. Here, we demonstrate that extracellular vesicles (EVs) produced by the corneal epithelium in response to exogenous NGF negatively affect the viability, proliferation, and migration of corneal epithelial cells. We found that EVs produced in response to NGF include an over‐enrichment in miR‐34a‐3p, while the same miRNA is under‐expressed in the cell lysate after NGF treatment. Conversely, treatment with a miR‐34a‐3p antagonist, miR34a‐i, induces a significant upregulation of endogenous NGF, suggesting a potential feedback pathway between miR‐34a‐3p and NGF. Furthermore, media conditioned with EVs produced in response to miR34a‐i treatment significantly enhances the proliferative response in the corneal epithelium. We identified bex3—encoding NGF receptor associated protein 1—as a potential target of miR‐34a‐3p in the NGF signaling pathway. These data highlight a potential intercellular negative feedback pathway induced by NGF treatment in the corneal epithelium.

## 1 Introduction

Nerve growth factor (NGF) is the prototypical neurotrophin—a class of proteins defined by their impact on neuronal cell development and health. (Levi‐Montalcini and Booker 1960; Levi‐Montalcini and Angeletti 1968) Neurotrophins like NGF are becoming increasingly recognized for their importance in wound healing processes in different tissues (Liu et al. 2021; El Baassiri et al. 2023; Schenck et al. 2017), including the human cornea (Kanu and Ciolino 2021). On the ocular surface, NGF secreted by the lacrimal gland, corneal and conjunctival epithelium, and trigeminal neurons plays a vital role in corneal wound healing, (You et al. 2000; Bonini 2002; Kanu and Ciolino 2021) and a recombinant human NGF (rhNGF) recently became the first FDA‐approved ophthalmic therapy for neurotrophic keratitis—a blinding disease caused by corneal nerve damage (Bonini et al. 2018; Bonini et al. 2018).

NGF exerts its intracellular effects via binding of one of two cell surface receptors, tropomyosin kinase receptor A (TrkA) or p75 neurotrophin receptor (p75NTR) (Kanu and Ciolino 2021). For example, NGF modulates the expression of numerous microRNAs (miRNAs)—small (19 to 25 nucleotides) (Rolle et al. 2016), noncoding RNAs that regulate gene expression, typically via interactions with the 3’ untranslated region (UTR) of target messenger RNAs (mRNAs) (O'Brien et al. 2018; Ha and Kim 2014). Since a single miRNA may have numerous mRNA targets, and since single mRNAs may be targeted by many miRNAs, miRNA signaling is complex and incompletely understood. For example, NGF has been reported to regulate miRNAs like miR‐21, miR‐20c, and miR‐93 in PC12 cells (Montalban et al. 2014), which each have myriad targets. Moreover, several members of the let‐7 family of tumor suppressor miRNAs—let‐7b, let‐7d, and let‐7i—are downregulated following NGF treatment (Abdolahi et al. 2022). Conversely, miRNAs like miR‐155 and miR‐455‐3p can target NGF mRNA (Abdolahi et al. 2022), thereby modulating NGF expression. Since miRNA signaling is highly cell‐ and context‐dependent, exploring the corneal epithelial cell‐specific NGF‐miRNA signaling is important.

In addition to intracellular miRNA signaling, NGF activates complex intercellular communication mechanisms, including endocrine, paracrine, and autocrine pathways (Sofroniew et al. 2001). Recently, extracellular vesicles (EVs)—nano‐scale, membrane‐bound particles released by all cells (Doyle and Wang 2019)—have emerged as critical mediators of the wound healing process in the cornea and other tissues (McKay et al. 2020; Samaeekia et al. 2018; Yeung et al. 2022; Narauskaitė et al. 2021). Moreover, recent studies have demonstrated that EVs secreted by NGF‐stimulated PC12 cells may modulate the neural differentiation process (Song et al. 2021). However, the functional contributions of EVs secreted by NGF‐stimulated corneal epithelial cells has not yet been described. EVs mediate wound healing via their cargo content as well as the spatiotemporal distribution thereof, and the quantity and content of EVs are altered during induced stress (i.e., during wound healing). For example, EVs secreted by the corneal epithelium are abundant in extracellular matrix proteins such as fibronectin and laminin (McKay et al. 2020). Moreover, microRNAs (miRNAs), such as miR‐381‐3p and miR‐199a‐3p, in corneal limbal epithelial cell derived EVs may be implicated in the proliferation and wound healing functions of these EVs (Verma et al. 2023). However, the mechanisms of these miRNAs are still being evaluated, and their relevance to exogenous growth factor therapy has not been extensively studied. As growth factors and related peptide‐based therapeutics continue to advance our wound healing options (Kanu and Ciolino 2021; Li et al. 2024; Fu et al. 2026; Ru et al. 2025), understanding their signaling effects in detail becomes increasingly important.

In the present study, we investigated how exogenous NGF influences intercellular communication in the corneal epithelium via EVs. Specifically, we characterized the effect of exogenous NGF on miRNA expression in corneal limbal epithelial cells and miRNA levels within their EVs. Further, we explored potential mechanistic connections between NGF‐responsive miRNAs and their functional effects.

## 2 Materials and Methods

### 2.1 Corneal Epithelial Cell Culture, NGF Treatment, and Transfection

An immortalized human corneal limbal epithelial (HCLE) cell line was used for cell culture studies (Gipson et al. 2003). HCLE cells were maintained in a humidified incubation chamber at 37°C and 5% CO_2_ and grown in complete media composed of Gibco Keratinocyte‐SFM (KSFM, Thermo Fisher Scientific) supplemented with 5 ng/mL recombinant human epidermal growth factor (rhEGF), 50 µg/mL bovine pituitary extract (BPE), and 1% penicillin–streptomycin (complete media). No serum or serum products were used in these studies. Prior to rhNGF treatment, cells were nutrient‐starved for 24 h in KSFM without rhEGF and BPE supplements (basal media). Media was then removed, cells were washed with PBS, and media was replaced with basal media supplemented with freshly prepared rhNGF solution (R&D Systems) in PBS or an equivalent volume of PBS (control).

### 2.2 HCLE Cell Viability, Proliferation, and Migration Assays

To assess cellular viability, HCLE cells were plated onto 96‐well plates at 1 × 10^4^ cells/well, left to adhere overnight, and treated with experimental conditions. After 24 h, cells were washed and then incubated in a staining solution [basal media supplemented with 8 µg/mL fluorescein diacetate (FDA) and 20 µg/ mL propidium iodide (PI)]. Cells were then visualized with a DMi8 fluorescence microscope (Leica Microsystems, Wetzler, Germany). For quantitative cellular viability assays, HCLE cells were seeded in 96‐well plates at a seeding density of 3 × 10^3^ cells/well. Cells were allowed to attach overnight, then underwent 24 h of nutrient starvation in basal media before being exposed to the experimental conditions. After 24 h of treatment, cells were washed then incubated in a solution of Invitrogen PrestoBlue Reagent (ThermoFisher Scientific) in basal media (1:10) for 1 h at 37°C. A plate reader was used to measure the fluorescence of the media at 560 nm excitation and 590 nm emission, and the percent reduction of PrestoBlue Reagent was determined per manufacturer instructions. Results were expressed relative to negative control in each experiment.

To assess cellular migration and proliferation, HCLE cells were seeded in 96‐well plates at a seeding density of 1 × 10^4^ cells/well and maintained in complete media. After achieving 80%–90% confluence, cells were nutrient starved for 24 h. Cells were typically 100% confluent after 24 h, following which a linear scratch was created in the center of the well using a 10 µL pipet tip. Cells were washed and then exposed to experimental conditions. Brightfield stereomicroscopic images were obtained using an EVOS XL Core microscope (ThermoFisher Scientific). Images were taken at 0‐ and 12‐h following scratch. Digital images were processed using ImageJ (version 1.54q, National Institutes of Health), which was used to measure the remaining wound area in each image.

### 2.3 Extracellular Vesicle Isolation and Characterization

Following 24 h of rhNGF treatment, cell‐conditioned media was collected from HCLE cells to isolate the released EVs using a differential centrifugation method. Media underwent centrifugation at 500 × g for 5 min, then 2000 × g for 5 min, then 13,000 × g for 15 min. Media was then concentrated using a 100 kDa MW filter (Centricon Plus‐70 centrifugal filter unit). The concentrated media then underwent ultracentrifugation at 150,000 × g for 70 min at 4°C (Beckman Coulter Optima LE‐80K ultracentrifuge). The pellet was washed and resuspended in PBS, followed by a second ultracentrifugation at 150,000 × g for 70 min at 4°C. The supernatant was removed, and the pellet was stored at −80°C until further use.

EVs were characterized according to recommendations for established guidelines (Welsh et al. 2024). EV concentrations and size distributions were determined using nanoparticle tracking analysis (NTA). Briefly, samples were diluted in sterile PBS to a final volume of 1 mL to achieve the appropriate measurement range for the NTA instrument (150−200 particles/frame). Samples were then added to the NTA instrument (NanoSight LM10; Malvern Panalytical; Malvern, UK) according to manufacturer instructions. Recordings were performed at 25 frames/second for 60 s each and repeated in triplicate.

EVs were visualized using transmission electron microscopy (TEM) as per a standardized protocol (Théry et al. 2006). Briefly, resuspended EVs were fixed in 2% paraformaldehyde and deposited onto Formvar‐coated nickel grids. After adsorbing, grids were washed with PBS, followed by staining with 1% glutaraldehyde for 5 min. Contrast was achieved using uranyl‐oxalate solution followed by a methyl cellulose‐uranyl acetate solution. Stained grids were imaged using a FEI Tecnai G2 Spirit transmission electron microscope (ThermoFisher FEI, Hillsboro, OR) at 100 kV accelerating voltage, interfaced with an AMT XR41 digital CCD camera (Advanced Microscopy Techniques, Woburn, MA) for digital image acquisition.

EVs were evaluated for the presence and absence of characteristic exosome markers using Exo‐Check Exosome Antibody Array according to manufacturer instructions. Briefly, protein concentrations were first determined using the bicinchoninic acid (BCA) protein assay (BioRad Laboratories, Hercules, CA). 50 µg of EVs were lysed using Lysis Buffer and labeled with Labeling Reagent, followed by column separation to remove excess Labeling Reagent. Membranes from the kit were incubated in labeled EV lysate along with blocking buffer overnight at 4°C. Membranes were washed, followed by incubation in Detection buffer and visualized by chemiluminescence (Syngene G:Box, India). Additionally, as a secondary quality control measure, EVs and pellets/supernatants produced during EV isolation were evaluated for positive and negative EV control markers using Western Blot using antibodies to GM130, calnexin, CD9, and CD63 (System Biosciences, Newark, CA).

Tetraspanin distributions at the individual EV level were evaluated using the ExoView R100 platform (NanoView Bioscience) (Hefley et al. 2022). Briefly, resuspended EVs were incubated on microarray chips (Leprechaun Exosome Human Tetraspanin Kit, Part #251‐1044, Unchained Labs, Pleasanton, CA, USA) overnight, followed by incubation with the fluorescent antibody cocktail provided in the kit (CD9, CD63, and CD81). The chips were then scanned using the ExoView R100 instrument and analyzed with the ExoView Analyzer Software.

### 2.4 MiRNA Isolation and Sequencing

miRNA from EVs or HCLE cells were extracted using the mirVana miRNA Isolation Kit (Life Technologies Corporation, Carlsbad, CA). Extracted miRNA concentration and purity were assessed using a NanoDrop Microvolume Spectrophotometer (ThermoFisher). miRNA isolates underwent library construction, and single‐end sequencing 50 bp on an Illumina Hiseq 4000 sequencing by LC Sciences (Houston, TX, USA). Bioinformatics analysis was performed by LC Sciences, and we performed further bioinformatic analysis using publicly available Gene Ontology (GO) functional databases and Kyoto Encyclopedia of Genes and Genomes (KEGG) pathway databases. Exploratory sequencing was used for hypothesis generation, and single biological replicates of each sample were sequenced, followed by qPCR testing and validation on multiple biological replicates. Single sample comparisons were performed and thus, p‐values represent single‐sample p‐values without multiple testing correction.

### 2.5 Cell Transfection

HCLE cells were plated in 24 wells and, at 70% confluency, were transfected with miR‐34a‐3p mimic using Lipofectamine 3000 transfection reagent for 48 h, following the manufacturer's protocol. After 48 h of transfection, conditioned medium was collected and cells collected using mechanical scraping for further processing.

### 2.6 MiRNA Quantification in EVs and HCLE Cells

Isolated EVs were collected from rhNGF‐treated or control cells and were lysed using lysis buffer provided with SeraMir Exosomes RNA amplification kit (System Biosciences). Exosomal RNA were isolated following the manufacturer's instructions. In HCLE cells, after NGF treatment or transfection, cells were lysed using Trizol (ThermoFisher). miRNAs were isolated using the mirVana Isolation kit following the manufacturer protocol. Following RNA isolation, RNA from EVs or cells were analyzed using Agilent Bioanalyzer following manufacturer instructions, which confirmed the presence of abundant small RNA in both cells and EVs. 25 ng of RNA were converted into cDNA using TaqMan Advanced miRNA cDNA synthesis kit (Applied Biosystems). Following cDNA synthesis, adaptor annealing reactions were performed and then RT reactions were performed using the kit and manufacturer protocol. miRNA specific primers were designed as listed in Table 1 (Integrated DNA Technologies) and universal reverse primer was used to analyze the expression of specific miRNA in the EVs. Gene‐specific PCR were performed using iTaq Universal SYBR Green Supermix (Bio‐Rad) for both EVs and cells, and U6 snRNA was used as internal normalization control for both EVs and cells. Amplification was performed on a Bio‐Rad CFX Connect Real‐Time PCR Detection System under the following cycling conditions: initial denaturation at 95°C for 20 s, followed by 40 cycles at 95°C for 1 s and 60°C for 20 s. All reactions were run in technical triplicate.

**TABLE 1:** Forward primers used for reverse transcription quantitative PCR.

Gene	Sequence
miR‐34a‐3p	TGGCAGTGTCTTAGCTGGTTGT
*Bex3*	GCCATACCCAATAGGCAGATCAA
*NGFR*	CCTCATCCCTGTCTATTGCTCC
U6	CGCAAGGATGACACGCAAATTC

### 2.7 Protein Quantification Using ELISA and Western Blot

NGF levels were determined by enzyme‐linked immunosorbent assay according to manufacturer's instructions (Invitrogen NGF beta Human ELISA Kit; ThermoFisher).

To evaluate the feedback response following inhibition of miR‐34a‐3p in HCLE cells, p75 neurotrophin receptor (p75NTR) protein expression was quantified by Western Blot (WB). Briefly, HCLE cells were transfected with miR‐34a‐3p antagomir (miR34a‐i), washed with PBS, and lysed with radioimmunoprecipitation assay (RIPA) buffer (10 mM Tris, 150 mM NaCl, 1% sodium deoxycholate, 1% Triton X‐100, 0.1% SDS, and 1 mM EDTA) supplemented with a protease inhibitor cocktail. Protein concentrations in cell lysate were determined using the Pierce BCA Protein Assay Kit (Thermo Fisher Scientific, Waltham, MA, USA). Equal protein amounts were separated on 4%–20% Mini‐PROTEAN TGX precast polyacrylamide gels (Bio‐Rad, Hercules, CA, USA), then transferred onto polyvinylidene fluoride (PVDF) membranes. Membranes were blocked for 1 h at room temperature in 5% skim milk prepared in PBST (PBS containing 0.1% Tween‐20), then incubated overnight at 4°C with primary antibody against p75NTR or GAPDH (Cell Signaling Technology, Danvers, MA, USA; Cat# 4201 or 51332, respectively). After washing three times with PBST, membranes were incubated for 1 h at room temperature with HRP‐conjugated anti‐rabbit IgG secondary antibody (Cell Signaling Technology, Danvers, MA, USA; Cat. 7074). Protein bands were detected using enhanced chemiluminescence reagents. Band intensities were quantified using ImageJ, and p75NTR levels were normalized to GAPDH as the loading control.

### 2.8 Argonaute‐miRNA Immunoprecipitation

miRNA targets in HCLE cells were validating with Argonaute‐RNA immunoprecipitation (Ago RIP), using miRNA Target IP Kit (Active Motif, Carlsbad, CA) according to the manufacturer's instructions. Briefly, HCLE cells were transfected with either miR‐34a‐3p mimic or no template control for 48 h. Cells were then washed, scraped, and lysed with lysis buffer. G‐coupled magnetic beads and pan‐Ago antibody were used to precipitate miRNA/mRNA complexes, while an isotype antibody was used as control. Argonaute proteins were then digested, and RNA from the precipitated complexes were purified using phenol:chloroform:isoamyl alcohol (25:24:1). Purified RNA was quantified and used for cDNA synthesis using iScript cDNA Synthesis Kit (Bio‐Rad) following the manufacturer's instructions. qPCR was performed using primers for miR‐34a‐3p, NGFR, and BEX3, using SYBR Green (Bio‐Rad) and CFX‐Connect Real Time System cycler (Bio‐Rad). Data were analyzed using Bio‐Rad CFX Manager 3.1 software and Microsoft Excel.

### 2.9 Statistical Methods

Data are expressed as mean ± standard error of the mean (SEM). Differences between groups were examined using statistical tests including student *t* test and Mann–Whitney test, based on normality which was assessed using Shapiro–Wilk test. Values of *p* < 0.05 were considered statistically significant.

## 3 Results

### 3.1 RhNGF Induced Differential miRNA Expression in Corneal Epithelial Cells

Exogenous recombinant human nerve growth factor (rhNGF) has been demonstrated to promote corneal epithelial wound healing in preclinical studies and has become widely adopted by clinicians to treat persistent corneal epithelial defects in patients with neurotrophic keratitis (Bonini et al. 2018; Bonini et al. 2018). We investigated how these beneficial effects may be contributed by differential expression of miRNAs in human corneal limbal epithelial (HCLE) cells. From our previous work (Kanu et al. 2024), we found that at a concentration of approximately 100 nM, rhNGF produces an optimal increase in HCLE cell proliferation at 24 h (i.e., the lowest concentration of rhNGF achieving maximal effect on HCLE cell proliferation, Figure S1). Therefore, we used this concentration and time point to identify NGF‐induced differential miRNA expression in HCLE cells.

**FIGURE 1:**
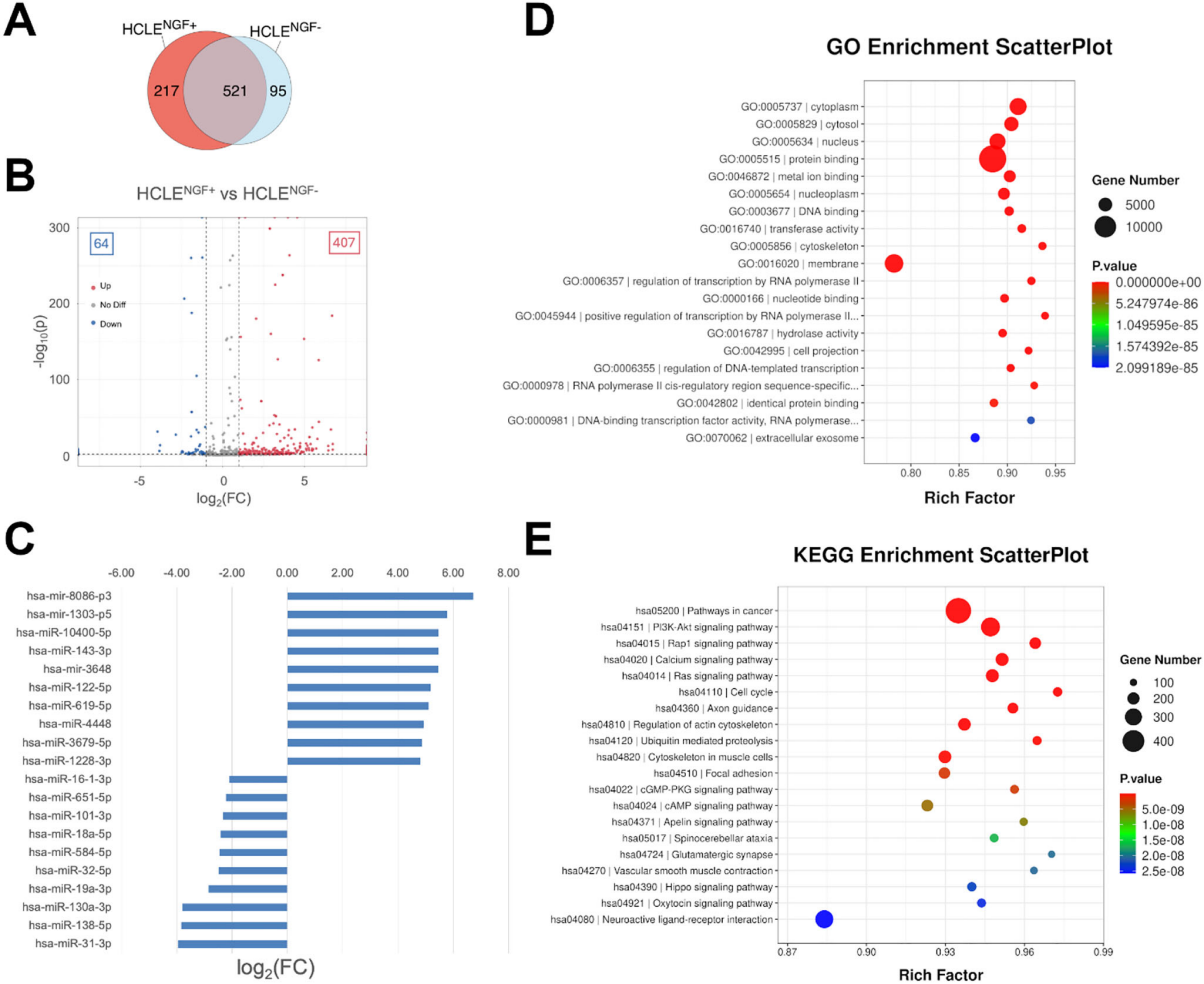
Small RNA sequencing revealed differentially expressed miRNAs in HCLE cells following treatment with 100 nM rhNGF in single biological replicates. (A) Venn diagram of all detected miRNAs in rhNGF‐treated HCLE cells (red) and controls (blue). (B) Up‐ (red) and down‐ (blue) regulated miRNAs rhNGF‐treated HCLE cells after 24 h. (C) Top 20 up‐ and down‐regulated miRNAs. (D, E) Bioinformatic enrichment analyses. Gene Ontology (GO, D) and Kyoto Encyclopedia of Genes and Genomes (KEGG, E) enrichment analysis of overrepresented pathways based on the differentially expressed miRNA in rhNGF‐treated HCLE cells.

Small RNA sequencing of single biological replicates of cell lysates (Figure 1, S2) following 24 h of treatment with either 100 nM rhNGF or an equivalent volume of phosphate‐buffered saline (PBS, control) revealed 833 unique miRNAs in HCLE cell lysates (Figure 1A), including 407 upregulated and 64 downregulated miRNAs (Figure 1B). The top ten up‐ and top ten down‐regulated miRNAs were identified (Figure 1C), and Gene Ontology (GO) and KEGG enrichment analyses were performed on the differentially expressed miRNAs. GO enrichment analysis revealed a significant enrichment in miRNAs related to protein binding, exosomes, and membranes (including those involved in exosome biogenesis, release, and uptake, including membrane pit‐coating proteins) (Figure 1D). KEGG enrichment analysis revealed significant differential expression of miRNAs involved in pathways related to cancer and phosphoinositide 3‐kinase‐protein kinase B (PI3K‐Akt) signaling—pathways involved in the regulation of cell growth, proliferation, and survival (Figure 1E). Therefore, KEGG and GO enrichment analyses suggest a potential role for rhNGF‐induced exosomes in mediating the cell proliferative response.

### 3.2 RhNGF Does Not Significantly Alter Physicochemical EVs in Corneal Epithelial Cells

**FIGURE 2:**
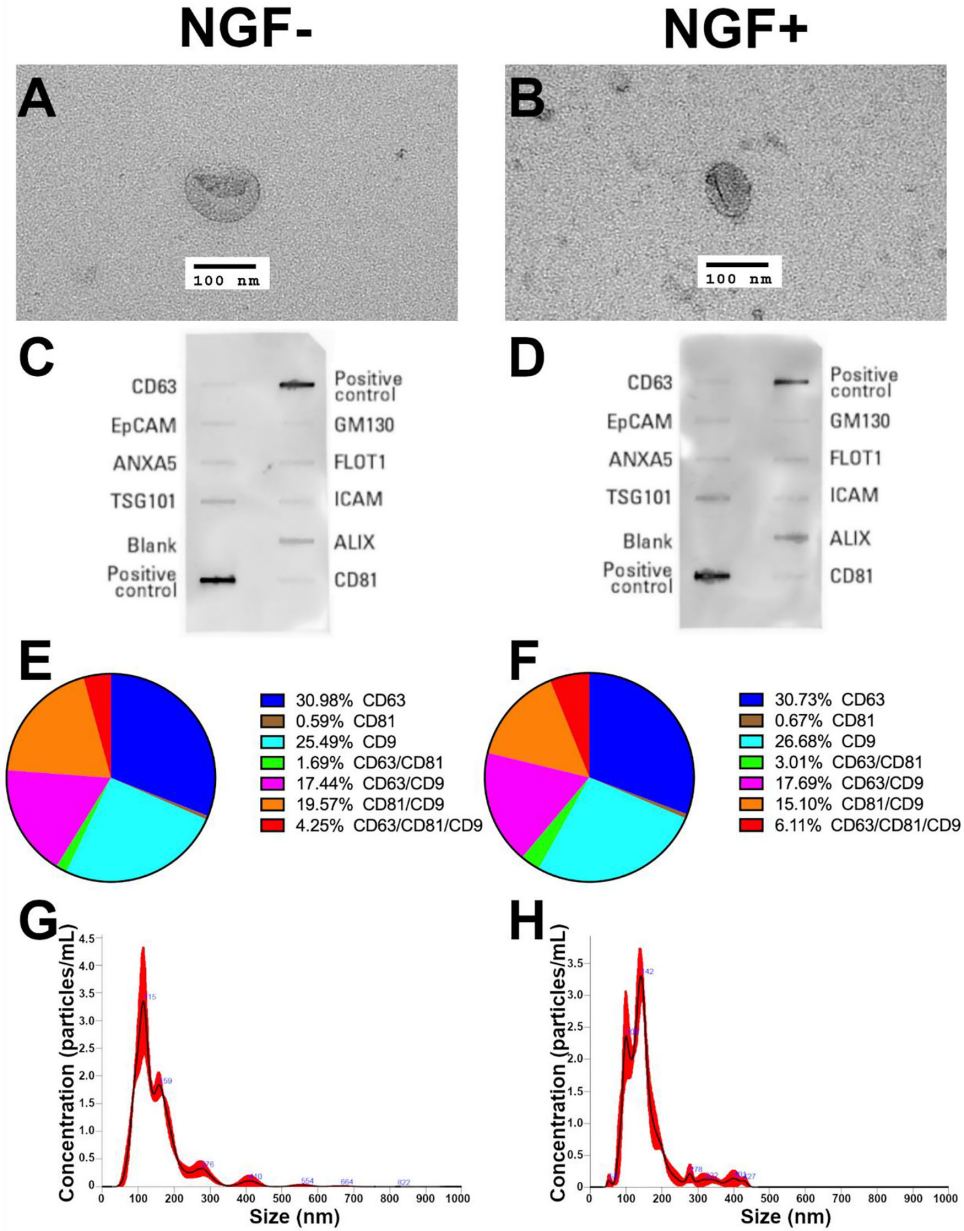
Physichochemical characterization of EVs produced by HCLE cells in response to rhNGF. (A, B) Transmission electron microscopy images of EVs produced by HCLE cells under control conditions (EV^NGF−^, A) or 24 h following treatment with 100 nM rhNGF (EV^NGF+^, B). (C, D) Exosome antibody arrays demonstrating that both EV^NGF−^ (C) and EV^NGF+^ (D) exhibited marker profiles consistent with established exosome markers; blots were loaded with equivalent amounts of protein. (E, F) Single‐EV level phenotyping of EV^NGF−^ (E) and EV^NGF+^ (F) based on colocalization of exosome tetraspanins CD63, C81, and CD9. (G, H) Representative nanoparticle tracking analysis of EV^NGF−^ (G) and EV^NGF+^ (H) revealed similar size distributions between the two sets of EVs.

Given the identification of differentially expressed miRNAs related to exosomes, we sought to characterize the EVs produced by HCLE cells in response to rhNGF. After 24 h, EVs were isolated from HCLE cells treated with either 100 nM rhNGF (EV^NGF+^) or PBS control (EV^NGF−^) using a differential ultracentrifugation method (Yeung et al. 2024). Transmission electron microscope (TEM) imaging revealed similar EV morphology between the two sets of EVs, including characteristic saucer shaped particles (Figure 2A,B). No difference in the shape of the EVs was observed. Both sets of EVs were Alix‐, EpCAM‐, and TSG101‐positive, consistent with established standards of EV biomarkers (Figure 2C,D). EVs from both conditions demonstrated similar tetraspanin compositions, EV^NGF+^ exhibiting a higher proportion of CD63/CD81+ EVs and a lower proportion of CD81/CD9+ EVs (Figure 2E,F). EV^NGF+^ and EV^NGF−^ were similar in size distribution [diameter 151.8 ± 11.9 and 156.2 ± 5.9 nm, respectively, (mean±SEM), *p* > 0.05, Figure 2G,H]. HCLE media following rhNGF treatment was more abundant in EVs compared to that of control cells (1.32 ± 0.16 relative number of particles per sample, *p* = 0.02). EVs were also measured for NGF content. While EV^NGF−^ contained undetectable levels of NGF by ELISA, EV^NGF+^ contained 6.02 pg per million EV particles.

### 3.3 EVs Produced by HCLE Cells in Response to rhNGF Reduce Corneal Epithelial Cell Proliferation and Migration at Supraphysiologic Levels

**FIGURE 3:**
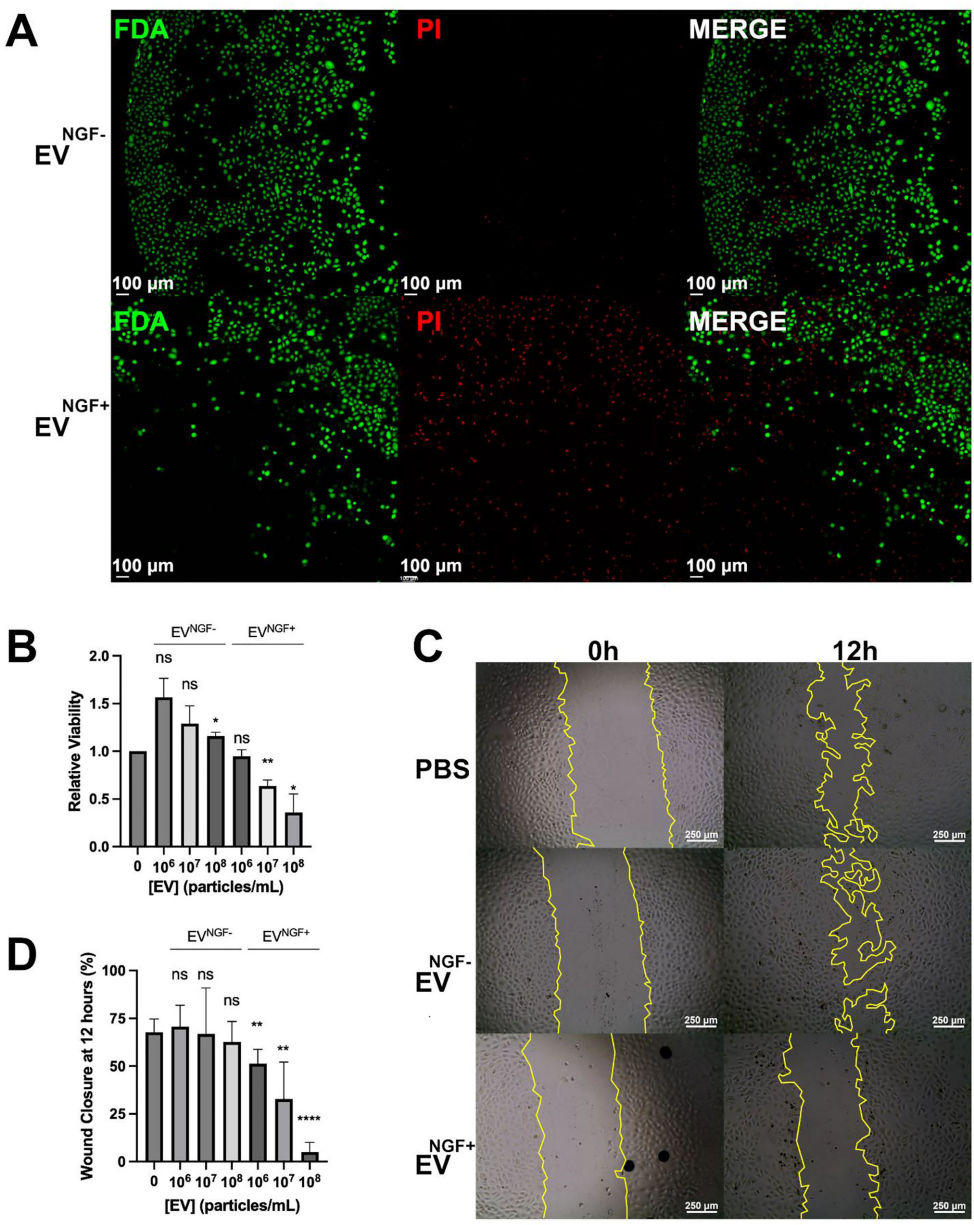
Functional effects of EVs produced by HCLE cells in response to rhNGF treatment. EVs were collected from HCLE cells after treatment with 100 nM rhNGF (EV^NGF+^) or control (EV^NGF−^) and added to treatment naïve HCLE cells. (A) Live/Dead assay following treatment with 10^7^ particles/mL of EV^NGF−^ or EV^NGF+^. Live cells stain with fluorescein diacetate (FDA, green) and dead cells stain with propidium iodide (PI, red). Scale bars indicate 100 µm. (B) Quantification of cell viability of HCLE cells was measured by resazurin‐based metabolic assay following supplementation with EV^NGF−^ or EV^NGF+^, or an equivalent volume of PBS control (*n* = 4). (C, D) The effect of EVs on wound healing was assessed using an in vitro scratch assay. A linear scratch was created in a confluent layer of HCLE cells, prior to supplementation with EV^NGF−^ or EV^NGF+^. (C) Representative micrographs of cells immediately following (0 h) and 12 h following injury (12 h). Scale bars indicate 250 µm. (D) Summary data of percent wound closure after 12 h under different experimental conditions (*n* = 6). Data are represented as mean±SEM; *p < 0.05; **p < 0.01; ****p < 0.0001; unpaired *t*‐test, all compared to [EV] = 0.

To understand the functional differences in EVs resulting from rhNGF treatment relevant to wound healing, we treated HCLE cells with EV isolates (EV^NGF+^ and EV^NGF−^, at equivalent concentrations) and determined the effect on cell viability, proliferation, and migration. Cell viability was assessed using a live‐dead assay, where incubation in media supplemented with isolated EV^NGF+^ (10^7^ particles/mL) (Liu et al. 2022) for 24 h resulted in significant cell death, while an equivalent concentration of EV^NGF−^ produced no such effect (Figure 3A). These data were quantitatively verified using a resazurin dye‐based fluorometric assay, as previously described (Kanu et al. 2024), and confirmed across a range of concentrations (10^6^, 10^7^, and 10^8^ particles/mL) (Liu et al. 2022). While EV^NGF−^ had a modest but statistically significant positive effect on HCLE cell viability at 10^8^ particles/mL (and no significant effect at lower concentrations), EV^NGF+^ produced a significant, dose‐dependent, negative effect on cell viability, with approximately 65% reduction in cell viability at 10^8^ particles/mL (Figure 3B). Cell proliferation and migration were assessed using an in vitro scratch assay (Kanu et al. 2024), in which EV^NGF+^ was found to significantly impair wound closure (Figure 3C,D). While EV^NGF−^ had no significant effect on the wound closure after 12 h at any tested concentration, EV^NGF+^ at all tested concentrations significantly inhibited wound healing in a dose dependent manner. Specifically, while untreated cells achieved on average 67.7% ± 7.0% wound closure after 12 h, cells treated with 10^6^, 10^7^, and 10^8^ particles/mL EV^NGF+^ achieved 51.2% ± 7.5%, 32.8% ± 19.3%, and 4.9% ± 5.2% wound closure in the same period of time. Finally, as a quality control measure, to assess quality of EV isolation and rule out organelle contamination as the cause of the negative functional effects exhibited by EV^NGF+^, we confirmed that EV^NGF+^ fractions lacked GM130 and calnexin cellular component markers (Figure S3).

To provide context to our findings, rather than exposing cells to the specific concentrations of EVs, in a separate assay, conditioned media and fractions thereof obtained directly from treated cells (i.e., taken from an equivalent volume of media and without undergoing 100 kDa filtration), and exposed directly to treatment‐naiive cells. In this assay, we found that the overall effect of the EV^NGF+^ and EV^NGF−^ on cell viability was not significant (Figure S4). Hence, our findings represent an exaggerated manifestation of the biological functions of EV^NGF+^ relative to physiologic conditions.

### 3.4 Expression Patterns of Small Noncoding RNA in HCLE Cell‐Derived EVs Following Treatment With rhNGF

**FIGURE 4:**
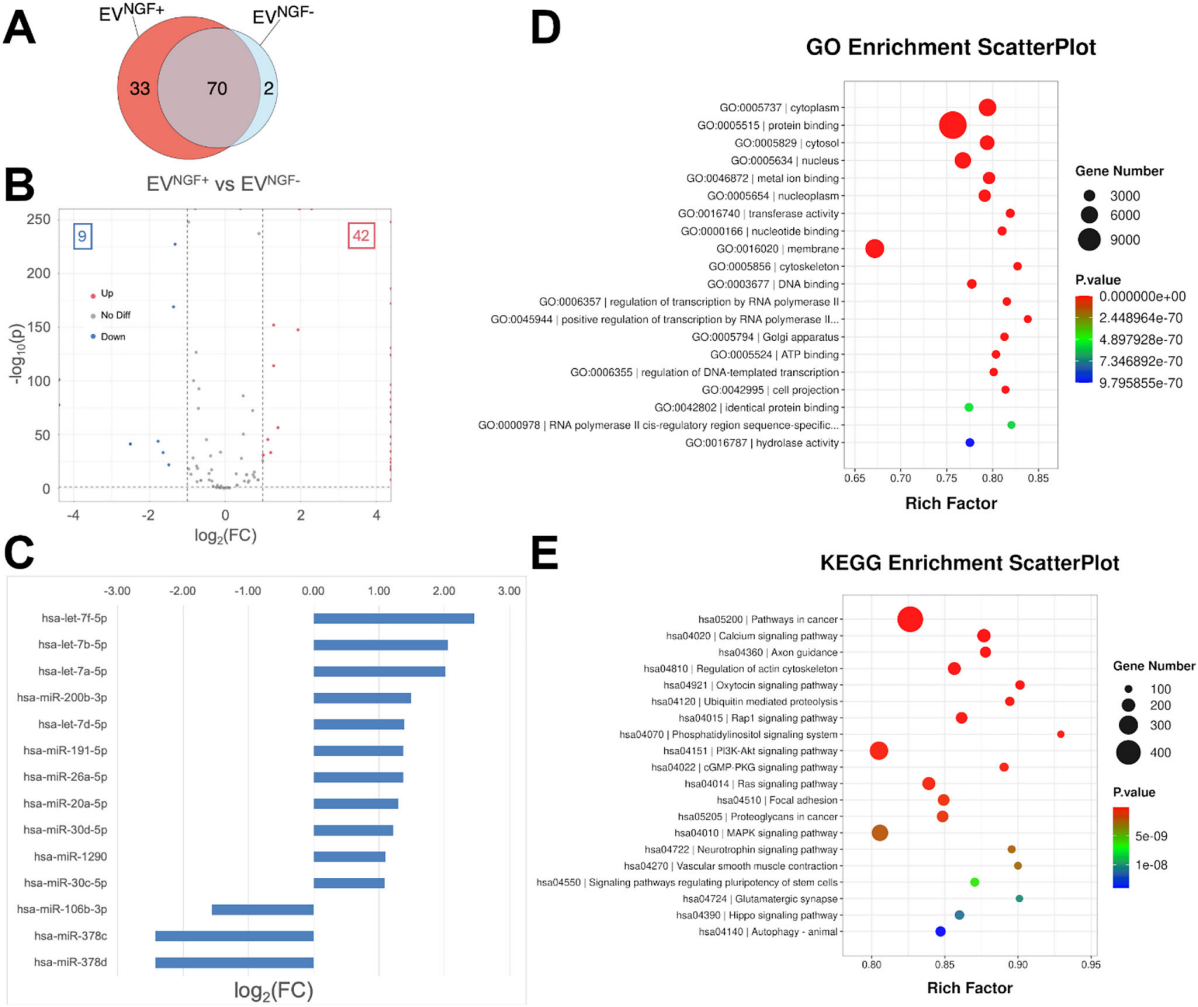
Small RNA sequencing revealed differentially expressed miRNAs in extracellular vesicles (EVs) produced by rhNGF‐treated HCLE cells in single biological replicates. (A) Venn diagram of all detected miRNAs in EVs obtained from rhNGF‐treated HCLE cells (red) and control cells (blue). (B) Up‐ (red) and down‐ (blue) regulated miRNAs in EVs obtained from 100 nM rhNGF‐treated HCLE cells after 24 h. (C) Top 14 up‐ and down‐regulated miRNAs. (D, E) Bioinformatic enrichment analyses. Gene Ontology (GO, D) and Kyoto Encyclopedia of Genes and Genomes (KEGG, E) enrichment analysis of overrepresented pathways based on the differentially expressed miRNA in HCLE‐derived EVs.

To elucidate potential mechanisms behind the negative effects of EV^NGF+^ on corneal epithelial cell proliferation, viability, and migration, we first used small RNA sequencing to explore the miRNA content of single biological replicate batches of EV^NGF+^ and EV^NGF−^ (Figure 4, S5). miRNA sequencing revealed 105 unique miRNAs (Figure 4A), including 42 upregulated and 9 downregulated miRNAs (Figure 4B). Upregulated miRNAs included several from the let‐7 family (Figure 4C), which is often considered to have tumor suppressor (i.e., anti‐proliferative) properties (Ma et al. 2021). GO enrichment analysis revealed a similar distribution of processes to that of the HCLE cell analysis (Figure 4D), including miRNAs relevant to protein binding and cell membranes. KEGG enrichment analysis revealed similar pathways to the HCLE cell sequencing analysis, including miRNAs relevant to pathways involved in cancer and PI3K‐Akt signaling, as well as mitogen‐activated protein kinase (MAPK) signaling (Figure 4E).

### 3.5 NGF's Anti‐Proliferative Effects Are in Part Mediated by miR‐34a‐3p

**FIGURE 5:**
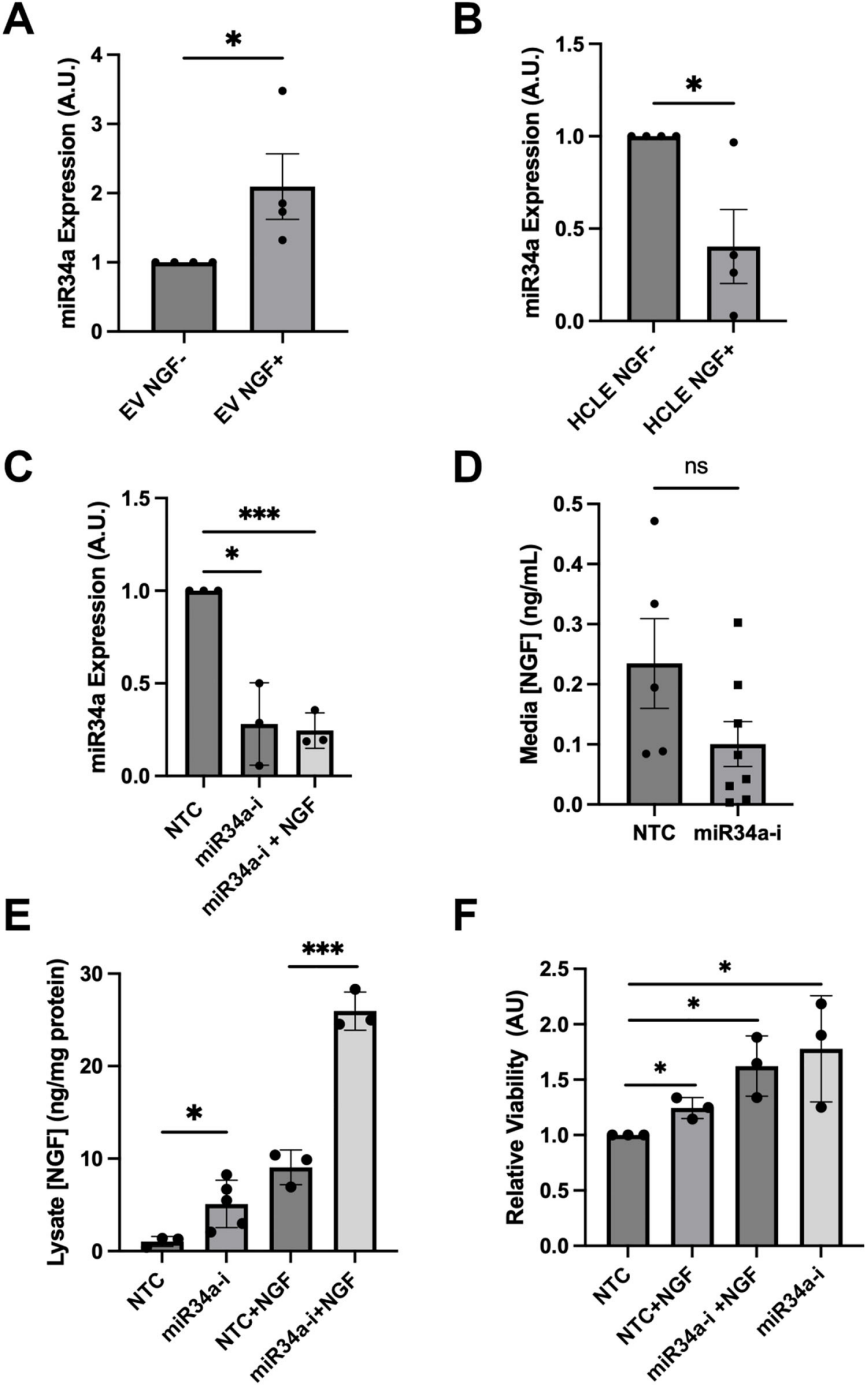
Interdependence of NGF and miR‐34a‐3p in HCLE cells. (A, B) miR‐34a‐3p levels in cell lysates (A) and EVs (B) produced by 100 nM rhNGF‐treated HCLE cells, compared to control (*n* = 4). (C) Validation of transfection efficiency of antagomir (miR34a‐i). miR‐34a‐3p levels are presented following transfection with no template control (NTC), miR34a‐i, and miR34a‐i followed by rhNGF. miR‐34a‐3p levels were normalized to U6 snRNA levels in each case (*n* = 3). (D, E) NGF levels in conditioned media (D, *n* = 6) and cell lysate (E, *n* = 3) following transfection with NTC or miR34a‐i, determined by ELISA. Intracellular NGF (lysate) levels are also presented following HCLE transfection with NTC or miR34a‐i followed by rhNGF. (F) Cell viability, measured by resazurin‐based metabolic assay, of HCLE cells following transfection with NTC or miR34a‐i, with or without subsequent supplementation with 100 nM rhNGF (*n* = 3). Data are represented as mean ± SEM; *p < 0.05; ***p < 0.001, Mann–Whitney test (**a, b, e**); *p < 0.05; ***p < 0.001; unpaired *t*‐test (**c, d, f**).

Real‐time polymerase chain reaction (qPCR) was used to validate differentially expressed miRNA in EVs. Given the prevalence of known tumor suppressor miRNAs in EV^NGF+^, such as those in the let‐7 family, we sought to quantitatively validate tumor suppressor miRNAs present in EV^NGF+^. While let‐7 miRNAs were some of the most overabundant in EV^NGF+^ in the sequencing results, real‐time polymerase chain reaction (qPCR) analysis of the EVs did not reveal statistically significant enrichment in let‐7. However, qPCR did reveal significant enrichment of another tumor suppressor miRNA, miR‐34a‐3p, in EV^NGF+^ [1.4 ± 0.3 fold change (FC), *p* = 0.03, Figure 5A]. In contrast, miR‐34a‐3p expression within HCLE cell lysates was significantly reduced following NGF treatment (0.4 ± 0.2 FC, *p* = 0.02, Figure 5B), suggesting a possible NGF effector molecule in HCLE cells. Given that miR‐34a‐3p was found to be over‐expressed in EV^NGF+^ by miRNA sequencing analysis and given that miR‐34a‐3p levels exhibited a directional enrichment consistency in qPCR analysis (i.e., reduction in cells and enrichment in EVs), we selected miR‐34a‐3p for further validation.

To further investigate potential interactions between miR‐34a‐3p and NGF, we first transfected HCLE cells with antagomiR‐34a‐3p (miR34a‐i) and confirmed a significant reduction in miR‐34a‐3p levels (0.28 ± 0.31 FC, *p* = 0.02, Figure 5C). Our data showed that rhNGF‐treated miR‐34a‐i‐treated cells had decreased expression of miR‐34a‐3p (0.20 ± 0.08 FC, *p* = 0.0003, Figure 5C). Furthermore, intracellular NGF was significantly increased in miR34a‐i–treated cells compared to controls (5.1 ± 2.6 vs. 1.0 ± 0.54 ng/mg protein, *p* = 0.03, Figure 5E). Interestingly, intracellular NGF levels following treatment with rhNGF were far greater in those cells transfected with miR34a‐i first (26.0 ± 1.2 ng/mg protein), compared to no template controls (NTC, 9.1 ± 1.1 ng/mg protein, *p* = 0.0005, Figure 5E).

These changes correlated with functional outcomes in the cell‐conditioned media. As we have previously demonstrated (Kanu et al. 2024), rhNGF treatment significantly improved HCLE cell viability (1.8 ± 0.5 FC, *p* = 0.04, Figure 5F), an output that correlates with cellular proliferation. We found that conditioned media from cells treated with rhNGF similarly enhanced cellular viability and proliferation compared to conditioned media from control cells (1.2 ± 0.1 FC in cell viability, *p* = 0.04, Figure 5F). EVs secreted by HCLE cells following transfection with miR34a‐i did not appear physicochemically distinct from controls (Figure S6). However, transfection with miR34a‐i produced an even greater increase in cell viability than did rhNGF treatment alone (1.6 ± 0.2 FC, *p* = 0.04, Figure 5F).

### 3.6 MiR‐34a‐3p May Modulate the NGF Signaling Pathway Through Post‐Transcriptional Regulation of bex3

**FIGURE 6:**
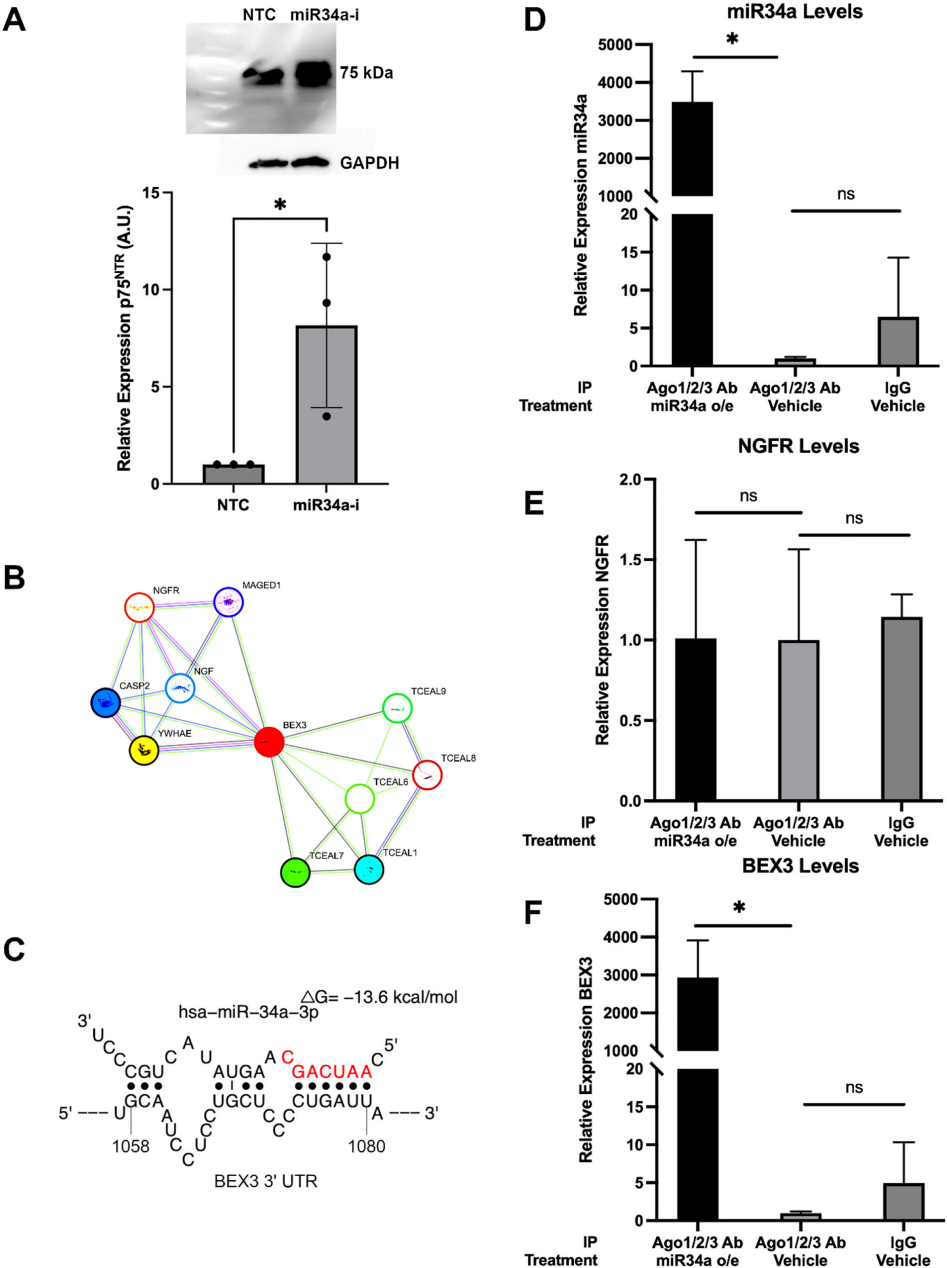
miR‐34a‐3p target identification within NGF signaling pathway. (A) Levels of p75 neurotrophin receptor (p75NTR) in HCLE cell lysates following transfection with miR34a‐i or no template control (NTC). p75NTR levels are reported normalized to U6 snRNA levels (*n* = 3). (B) Interaction network diagram of one of the predicted targets of miR‐34a‐3p, BEX3, demonstrating interactions between BEX3, NGF, and NGFR (also known as p75NTR). (C) Predicted 7mer‐A1 seed match between BEX3 3’ UTR and miR‐34a‐3p. (D–F) miR34a‐3p target identification using Argonaute RNA immunoprecipitation following transfection of HCLE cells with miR‐34a‐3p mimic. Depicted are levels of miR‐34a‐3p (A), NGFR (B), and BEX3 (C) co‐precipitated with Argonaute protein (*n* = 3). Data are represented as mean±SEM; *p < 0.05, unpaired t‐test.

Given the influence of miR‐34a‐3p on NGF levels, we sought to identify a potential feedback loop. In addition to increased NGF levels, we found that inhibition of miR‐34a‐3p resulted in significantly increased expression of p75 neurotrophin receptor (p75NTR, also known as NGFR) in HCLE cells (*p* = 0.04, Figure 6A). Using the online tool TargetScan (http://www.targetscan.org/) we identified potential targets of miR‐34a‐3p. Despite increased protein expression in our studies, neither NGF nor NGFR genes are predicted targets of miR‐34a‐3p. However, one of the reported miR‐34a‐3p targets is NGF Receptor Associated Protein 1 (NGFRAP1, or BEX3), a protein which is involved in NGF signaling as an adapter for p75NTR (Mukai et al. 2000). Interactions between NGF, BEX3, and p75NTR were visualized using the STRING database (Szklarczyk et al. 2023) (Figure 6B).

A 7mer‐A1 seed match (Figure 6C) was identified within the 3’ UTR region of the BEX3 mRNA, and verified with the online miRNA binding site prediction software Sfold STarMirDB (http://sfold.wadsworth.org/starmirDB.php). To validate this target prediction, we performed human argonaute (Ago) protein RNA immunoprecipitation (Ago RIP) in cells transfected with miR‐34a‐3p mimic. As expected, Ago proteins immunoprecipitated from miR‐34a‐3p overexpressed HCLE cells were found to be highly bound to miR‐34a‐3p (*p* = 0.01, Figure 6D), while NGFR did not co‐precipitate significantly with Ago proteins (*p* = 0.99, Figure 6E). However, *bex3* was found to co‐precipitate with Ago proteins in miR‐34a‐3p overexpressing cells (*p* = 0.04, Figure 6F), suggesting that miR‐34a‐3p may regulate *bex3* expression at the post‐transcriptional level.

## 4 Discussion

Our data show that EVs produced by the corneal epithelium in response to exogenous NGF treatment may possess contents that contribute negatively to corneal epithelial cell health and wound healing processes. Specifically, we found an overabundance of miR‐34a‐3p within these EVs—the inhibition of which greatly enhances NGF proliferative effects.

NGF is well known to produce pro‐survival effects in neuronal cells, primarily through the TrkA receptor, including via activation of phosphatidylinositol 3‐kinase (PI3K)/Akt signaling (Mnich et al. 2014; Szegezdi et al. 2008). Moreover, extensive in vitro and in vivo study has demonstrated NGF as a pro‐survival factor in the corneal epithelium and a potent stimulator of corneal wound healing (Lambiase et al. 2007; Lambiase et al. 2009; Blanco‐Mezquita et al. 2013; Lambiase et al. 2000). However, NGF signaling is complex and may involve opposing pathways depending on the context of the cell. For instance, NGF signaling through p75NTR mediates opposing effects on cell survival and apoptosis, depending on cell type (Lu et al. 2005; Hickman et al. 2018), and may induce pro‐apoptotic signaling via JNK and NF‐κB pathways (Micera et al. 2012; Gu et al. 2025; Kuner and Hertel 1998). Moreover, as expected from a growth factor with numerous, at times opposing, and highly cell context dependent, activated pathways. For instance, in the context of cornea, NGF may induce corneal neovascularization (Seo et al. 2001), and NGF‐p75NTR signaling may be implicated in one of the significant adverse effects of topical NGF therapy—pain (Obata et al. 2006). Therefore, understanding the complex signaling processes is imperative when using NGF as a therapy.

As our results support, EVs are increasingly being recognized as important agents of intercellular communication and signaling pathways. For example, EVs released by various cells including corneal epithelial cells and stromal keratocytes have proven to be important mediators of the complex process of corneal wound healing (McKay et al. 2020; Han et al. 2017; Desjardins et al. 2022). Moreover, EVs collected from regenerating corneal epithelium has recently been used as a therapy to promote corneal wound healing (Rosenquist Lybecker et al. 2025). However, to our knowledge, the EVs produced by the cornea in response to growth factors like NGF have not been extensively studied. Interestingly, our study found that while EVs derived from HCLE cells at a physiologic state did not significantly impact HCLE cell viability, proliferation, or migration, even low concentrations of EV^NGF+^ produced significant inhibition of these processes. We confirmed that these changes were not due to an elevated level of NGF packaged into the EVs, as the effective NGF concentration within the EVs was still far lower than a therapeutic concentration. Our study evaluated the functional effects of HCLE‐derived EVs at concentrations between 10^6^ and 10^8^ particles/mL. While reported EV concentrations are highly method‐dependent, we chose this range to include reported concentrations in various body fluids, including tears and blood (Johnsen et al. 2019; Lee et al. 2024). At these levels, EVs obtained from control HCLE cells—EV^NGF−^—seemed to positively impact cell viability. In contrast, EV^NGF+^ reduced both cell viability and migration in a dose‐dependent manner. Importantly, however, the concentrations of EVs used in our study are orders of magnitude greater than the physiologic levels experienced by HCLE cells in culture. This is because the EV concentration in media is quite low compared to biofluids (e.g., plasma and tears), thus requiring large volumes of media and high degrees of concentration for experimental analyses (Gardiner et al. 2016; Guerreiro et al. 2018). Thus, the concentrations used in our experimentation likely exaggerate the functional effects, and the relevance to in vivo systems is beyond the scope of the study. Indeed, at the physiological concentrations produced by the cells in our in vitro system, the EVs had no demonstrable effect on cell viability.

Interestingly, our small RNA sequencing analysis of HCLE cells revealed significant changes in expression of RNA related to exosomes. While little is known about the influence of NGF on EV production in the corneal epithelium, other growth factors have been found to influence EV production in different tissues—specifically, epidermal growth factor (EGF) modulates renal tubular cell EVs (Zhou et al. 2017) and fibroblast growth factor influences the EV production of hippocampal neurons (Kumar et al. 2020). Still, the mechanisms dictating the changes in EV contents and release in response to growth factors remains poorly understood.

To understand the mechanisms behind the negative functional effects of EV^NGF+^ on HCLE cells, we studied the miRNA content of the EVs. EVs mediate their effects through their various contents, including proteins and miRNAs, and the spatiotemporal distribution thereof (Lee et al. 2024; Kolenc et al. 2025). Given that single miRNAs may have numerous cellular targets and may therefore affect broader cellular responses compared to single proteins, we chose to focus on the miRNA content of the EVs. Interestingly, small RNA sequencing suggested an enrichment of several tumor suppressor miRNAs in EV^NGF+^. Specifically, several miRNAs in the let‐7 family of tumor suppressor miRNAs (Boyerinas et al. 2010) were overexpressed in EV^NGF+^ compared to EV^NGF−^. Our sequencing data was generated from single biological replicates and can thus only be considered exploratory in nature rather than representing a rigorous quantification. Single miRNA and biological pathway enrichment analyses therefore must be interpreted with caution, and further validation of these findings is necessary. Furthermore, due to generally low abundance of miRNA, differing normalization strategies, and variable primer efficiency–among other factors–correlation between sequencing data and quantitative PCR is challenging (Sampathkumar et al. 2022; Hong et al. 2021). Hence, the preliminary sequencing data prompted us to quantitatively evaluate for other known tumor suppressor miRNAs in the EVs, such as miR‐34a‐3p.

The miR‐34 family of miRNAs is well documented to mediate neuronal development and differentiation through interactions with NGF‐induced signaling pathways (Jauhari et al. 2018; Aranha et al. 2011). Specifically, NGF induces increased miR‐34a production, and miR‐34a appears to be important in suppressing cell proliferation in mature neurons (Jauhari et al. 2018). Interestingly, our study found that NGF induced a reduction in miR‐34a‐3p expression within corneal epithelial cells, while simultaneously increasing the abundance of miR‐34a‐3p within corneal epithelial cell‐derived EVs. While the mechanisms guiding this differential expression were not tested in this study, several mechanisms may explain these findings. Given the importance of miR‐34a in NGF signaling, selective packaging of miR‐34a‐3p into EVs may represent a method by which NGF exerts paracrine signaling, potentially as a negative feedback signal. For example, NGF may act through EV miRNA sorting factors like HNRNPA2B1 to guide selected miRNA into EVs (O'Grady et al. 2022). As we found indirectly that miR‐34a‐3p inhibits endogenous NGF production in HCLE cells, EV packaging of miR‐34a‐3p may represent a mechanism to modulate the local tissue response to an overabundance of NGF. Alternatively, although the EVs studied were likely not significantly comprised of apoptotic bodies based on their diameters, miR‐34a‐3p may be packaged into EVs as part of a waste removal process (Lee et al. 2024). Investigation into the mechanisms guiding the differential expression of miR‐34a‐3p and other miRNA cargo in EV^NGF+^ is warranted.

The mechanisms by which the miR‐34 family might modulate NGF signaling have not been fully described. We performed Argonaute RNA immunoprecipitation (Ago‐RIP) to identify potential targets of miR‐34a‐3p and found that *bex3* (NGFRAP1) co‐precipitates with over‐expressed miR‐34a‐3p. *Bex3* belongs to the Bex (Brain‐Expressed X‐linked) family of genes, which are all localized on the X chromosome and exhibit high levels of expression in the brain (Alvarez et al. 2005). While *bex3* was initially referred to as the p75‐associated death executor (NADE) due to its involvement in p75NTR‐driven, NGF‐induced apoptosis pathways (Mukai et al. 2000; Mukai et al. 2002), it has also been found to play an important role in promoting cell survival and neurite outgrowth in neurons (Roux 2002; Chen et al. 2009). Hence, while its exact roles remain convoluted, BEX3 protein has been demonstrated to promote TrkA production via associating with the *trkA* promoter region (Calvo et al. 2015). Furthermore, *bex3* silencing results in increased apoptosis in several neuronal cell types (Calvo et al. 2015). Our work suggests that BEX3 may play an important role in mediating NGF signaling in the corneal epithelium via an EV‐associated miR‐34a‐3p induced response to NGF. Investigation into the mechanisms guiding post‐transcriptional regulation of bex3 by miR‐34a‐3p is warranted.

In summary, this work describes a potential new mechanism by which NGF influences intercellular communication between cells, specifically via EV‐associated miR‐34a‐3p. EVs produced by corneal epithelium in response to exogenous NGF may have a negative regulatory mechanism that is in part mediated through miR‐34a‐3p and the post‐transcriptional regulation of *bex3* expression. Further investigation into EV‐mediated intercellular growth factor signaling pathways is warranted to better understand and optimize growth factor‐based therapies.

## Author Contributions

**Levi N. Kanu**: conceptualization, investigation, funding acquisition, writing – original draft, methodology, validation, visualization, writing – review and editing, software, formal analysis, project administration, data curation, supervision, resources. **Amit Chatterjee**: conceptualization, investigation, writing – review and editing, methodology, software, formal analysis. **Mohamed Y. Mahmoud**: methodology, writing – review and editing, data curation. **Vincent Yeung**: methodology, data curation, writing – review and editing. **Sushma V. Mudigunda**: methodology, writing – review and editing, data curation. **Amy E. Ross**: methodology, data curation, writing – review and editing. **Brenna Hefley**: methodology, formal analysis, data curation, validation, writing – review and editing, visualization. **Dimitrios C. Karamichos**: methodology, data curation, validation, visualization, writing – review and editing, resources. **Joseph B. Ciolino**: resources, methodology, writing – review and editing, funding acquisition.

## Funding

This work was supported by the US National Institutes of Health (NIH) grants K08EY037382 (LNK), K99EY037781 (AC), R01EY005665 (JBC), and P30EY003790 (Schepens Eye Research Institute Core).

## Conflicts of Interest

The authors report no conflicts of interest.

## Supporting information




**Supporting Information**: jex270192‐sup‐0001‐FigureS1‐S6.docx

## Data Availability

The datasets generated in the current study are available upon reasonable request from the corresponding author.
