# Supplementary material for: Nerve Growth Factor Treatment and the Corneal Epithelium: Extracellular Vesicle‐Mediated Signaling
Source: J Extracell Biol. 2026 Sep 17;5(9):e70192. doi: 10.1002/jex2.70192 (PMC13586549; doi:10.1002/jex2.70192)
Supplement: Supplementary file 1 [file JEX2-5-e70192-s001.docx]

Figure S1. Optimization of rhNGF concentration for viability assays. Serial dilutions of fresh rhNGF (0 to 1000 nM) were added to HCLE cells and the response after 24 hours was measured by resazurin-based metabolic assay. Media supplemented with epidermal growth factor and bovine pituitary extract (complete media, CM) served as a positive control. 100 nM was the lowest concentration achieving the highest level of relative cell viability, and higher concentrations did not produce significant additional positive effect.

Figure S2. Quality control and small RNA sequencing characterization of HCLE cells in response to rhNGF. (A, B) Quality scores of small RNA isolated from HCLE cells under control conditions (HCLE^NGF-^, A) or 24 hours following treatment with 100 nM rhNGF (HCLE^NGF+^, B). (C–F) Distribution of small RNA subtypes identified by sequencing of HCLE^NGF-^ and HCLE^NGF+^, displayed as percentages of unique RNAs (C, E) or total RNAs (D, F). (G, H) Length distributions of small RNAs identified by sequencing of HCLE^NGF-^ and HCLE^NGF+^, displayed as percentages of unique RNAs (G) or total RNAs (H).

Figure S3. Western Blot analysis of positive and negative quality control markers of isolated EV^NGF+^. Samples of EVs produced by HCLE cells following treatment with 100 nM rhNGF (EV^NGF+^) were compared alongside the pellet and supernatants obtained during isolation of the EVs. Samples were tested for Golgi (GM130) and endoplasmic reticulum/whole cell (calnexin) proteins, as well as positive EV control markers (CD63 and CD9).

Figure S4. Conditioned media components functional effects on HCLE cell viability. Conditioned media from cells treated with 100 nM rhNGF (“NGF”) or an equivalent volume of PBS (Control) were separated into components (supernatant and EV fractions) and added to treatment-naïve HCLE cells. Cell viability was measured by resazurin-based metabolic assay. A fresh treatment of 100 nM rhNGF served as a positive control. **p*<0.05; ***p*<0.01.

Figure S5. Quality control and small RNA sequencing characterization of EVs isolated from HCLE cells in response to rhNGF. (A, B) Quality scores of small RNA isolated from EVs produced by HCLE cells under control conditions (EV^NGF-^, A) or 24 hours following treatment with 100 nM rhNGF (EV^NGF+^, B). (C–F) Distribution of small RNA subtypes identified by sequencing of EV^NGF-^ and EV^NGF+^, displayed as percentages of unique RNAs (C, E) or total RNAs (D, F). (G, H) Length distributions of small RNAs identified by sequencing of EV^NGF-^ and EV^NGF+^, displayed as percentages of unique RNAs (G) or total RNAs (H).

Figure S6. Physichochemical characterization of EVs produced by HCLE cells following transfection with miR-34a-3p inhibitor (miR-34a-i). in response to rhNGF. (A, B) Single-EV level phenotyping of control (A) and miR-34a-i–treated cell–derived (B) EVs based on colocalization of exosome tetraspanins CD63, C81, and CD9 revealed similar tetraspanin distributions. (C, D) Representative nanoparticle tracking analysis (NTA) of control EVs (C) and miR-34a-i–treated cell–derived (D) EVs revealed similar size distributions between the two sets of EVs. (E, F) NTA results of control and miR-34a-i–treated cell–derived EVs revealed similar average particle diameters (E) and concentrations (F).
